# Ag-CuO/epoxy hybrid nanocomposites as anti-corrosive coating and self-cleaning on copper substrate

**DOI:** 10.1038/s41598-023-46533-x

**Published:** 2023-11-07

**Authors:** Amal M. Abdel-karim, Yousra M. Ahmed, Mai M. El-Masry

**Affiliations:** 1 Physical Chemistry Department, National Research Centre, 33 El Bohouth Street, Dokki, Giza, 12622 Egypt; 2 Civil Engineering Department, Higher Engineering Institute, Thebes Academy, Cairo, 11434 Egypt; 3 Basic Science Department, Higher Engineering Institute, Thebes Academy, Cairo, 11434 Egypt

**Keywords:** Chemistry, Engineering

## Abstract

This study aims to prepare Ag-CuO nanoparticles and assess their efficiency in protecting the copper substrate. The prepared Ag-CuO nanoparticle was characterized using, Fourier-transform infrared spectroscopy (FTIR), X-ray diffraction (XRD), scanning electron microscope/energy-dispersive X-ray (SEM/EDX), and transmission electron microscope (TEM). The anticorrosion performance of the epoxy coatings containing various weight percentages of Ag-CuO nanoparticles was evaluated in 3.5 wt% NaCl solution using potentiodynamic polarization (PDP), and electrochemical impedance spectroscopy (EIS) techniques. The results showed that corrosion potential shifted from − 0.211 V for uncoated copper to − 0.120 V for 5.0 wt% Ag-CuO/epoxy hybrid nanocomposite. Electrochemical measurements indicated that the coating 5.0 wt% coating exhibited excellent inhibiting properties with an efficiency of 99.9%. Wettability and mechanical properties were measured for both uncoated and coated copper substrates. The contact angle for 5.0 wt% coating is equal to 104° enhancing the hydrophobic character of the surface. The study clearly establishes that the hybrid composite has a significant potential for protecting the copper substrate.

## Introduction

Corrosion is one of the most common issues affecting metal components, and it is crucial to mitigate the deterioration of metals. Several techniques are employed to reduce corrosion, including cathodic or anodic protection^1^, inhibitors^2–4^, and coatings^5–8^. Extending the life cycle of metals in various environments has been the focus of numerous research studies aimed at developing effective methods safeguarding metals from corrosion. The advancement of nanotechnology has led to the synthesis of new materials with enhanced properties^9^. Efficient polymeric coatings^10^ and the hybrid (inorganic–organic) nanocomposites coating are excellent examples of a protective coating for metals^11–14^.

Copper and its alloys have found widespread use in numerous industrial applications^15^. The corrosion resistance of copper and its alloys is outstanding in pure air and drinking water. However, in the presence of impurities or aggressive ions such as chloride along with water and oxygen^16^, the corrosion rate increases, often resulting in localized corrosion or pitting. This can lead to alterations in the surface color and the morphology, potentially affecting the performance of system made from copper^17^.

Additionally, the corrosion products can be released into the environment, leading to pollution and changes in its appearance and properties. Hence, protecting copper is a crucial goal, with the current focus on finding cost-effective, high-efficiency coatings suitable for aggressive environment**.** Organic coatings containing chromium or lead are strictly prohibited due to their adverse effects on the human health and environment. Polymeric composite materials have garnered significant attention due to their flexibility, ease of processing, and high excellent corrosion protection.

In a neutral chloride solution, Guenbour et al.^18^ reported that poly (2-aminophenol) coating provides effective protection for copper. Brusic et al.^19^ discovered that spin-coated polyaniline film exhibited excellent protective performance on copper. Patil et al.^20^ conducted a study electrochemically synthesized poly(o-anisidine) for the protection of copper in 3.5 wt% NaCl. Özyılmaz et al. deposited polyaniline on the copper substrate using a supporting electrolyte (sodium oxalate)^21^.

One of the methods involves using metal and metal oxides nanoparticles as anti-corrosive for copper. Among the metal and metal oxides NPs that have been studied for this purpose, silver, silver oxide, copper, and copper oxide have exhibited significant potential in various applications due to their excellent physical and electrical properties^22^.

Silver and copper oxides are known for their high corrosion resistance, potent antibacterial effects, and low cost-effectiveness. However, both silver and copper oxide have certin drawbacks, including poor adhesion to copper substrate, limited stability under acidic or alkaline conditions, and a tendency to agglomerate. To address these limitations, epoxy resin has been employed as a binder or a matrix to enhance the adhesion and stability of Ag and copper oxide coatings on copper^23^.

Epoxy polymer coating are known for their scratch resistance and strong adhesion to various metal substrates^24^. However, epoxy coatings alone may not provide sufficient protection for metal and can deteriorate under prolonged exposure to environmental conditions. To address this studies have focused on the fabricating and characterization of epoxy-based coatings that incorporate metal oxide NPs for corrosion protection purposes.

For instance, the incorporation of graphene Gr NPs into a nontoxic epoxy polymer can serve as an environmentally friendly corrosion inhibitor^25^ aligning with the development of the “green chemistry” concept in the field of corrosion**.** Additionally, the utilization of nanoscale MgO in combination with epoxy has been known to enhance the performance of epoxy coating in saline solutions, achieving an efficiency of 93.7%^26^. Foyet et al. observed that the corrosion rate of epoxy coating containing either 1% or 1.25 volume fraction of carbon black (CB) is 10 times compared to a pure epoxy coating on Aluminum substrate^27^.

Nazarzade et al. synthesized CuO/epoxy composite coating by blending CuO nanoparticles with epoxy resin using a mechanical stirrer. They observed that the coating exhibited a smooth surface and a uniform distribution of CuO NPs^28^. In a similar vein Mousaa et al. proposed that the optimal content of CuO NPs in the epoxy resin should be 10 wt%. This suggests that the optimal loading of metal oxide NPs is contingent upon their size, shape, and interaction with the epoxy resin^29^.

The anti-corrosion properties of a material are also closely linked its hydrophobicity and self-cleaning characteristics of its surface, primarily assessed through the contact angle. Achieving self-cleaning surface properties for metals typically involves applying suitable coating materials that posses hydrophobic, antibacterial, or photo-degradation capabilities^30,31^.

Copper metal has garnered significant attention in hydrophobic applications, while Silver NPs have demonstrated exceptional antibacterial activity. Nanocomposites such as Ag with metal oxides (e.g. Ag/CuO) have shown promising results.

The objective of this study was synthesize Ag-CuO NPs using a cost-effective co-precipitation technique and characterizes them through XRD, FT-IR, and TEM analysis.

Subsequently, the Ag-CuO/epoxy hybrid nanocomposite was created via the solid-state reaction method, involving the incorporation of varying weight percentages (wt%) of Ag-CuO NPs into an affordable epoxy matrix. A comparative analysis between Ag-CuO/epoxy and pure epoxy was conducted to assess the advantage of incorporating NPs within the epoxy matrix. The anti-corrosion performance of the Ag-CuO/epoxy hybrid nanocomposites on copper substrates was evaluated using potentiodynamic polarization (PDP) and electrochemical impedance spectroscopy (EIS) techniques in a 3.5 wt% NaCl solution. The surface analysis was achieved through scanning electron microscopy (SEM), and water contact angle measurements were performed to further characterize the coatings.

## Experimental

### Materials

In this study, analytical graded mineral precursors including Silver nitrate AgNO_3_, Copper nitrate Cu (NO_3_)_2_0.6H_2_O, Sodium hydroxide pellets (NaOH), and Sodium Chloride were procured from Merck, Egypt. Epoxy resin (KEMAPOXY 150 JM) and the hardener HY 956 were obtained from (CMB Egypt) Chemicals for Modern Building Company; the resin samples were prepared with a mixed ratio of 3:1 (resin: hardener) by weight.

The Copper sheet (99% purity) measuring 1 cm × 1 cm and 1 mm in thickness was sourced from a local market in Cairo, Egypt. These sheets underwent a polishing process to achieve mirror finish and were subsequently abraded using emery papers with various grades (320, 400, 800, 1000, 1200, and 2000) to remove any oxide layer. Following this, they were rinsed with distilled water and degreased with acetone.

### Nanoparticle’s preparation

**Figure 1:**
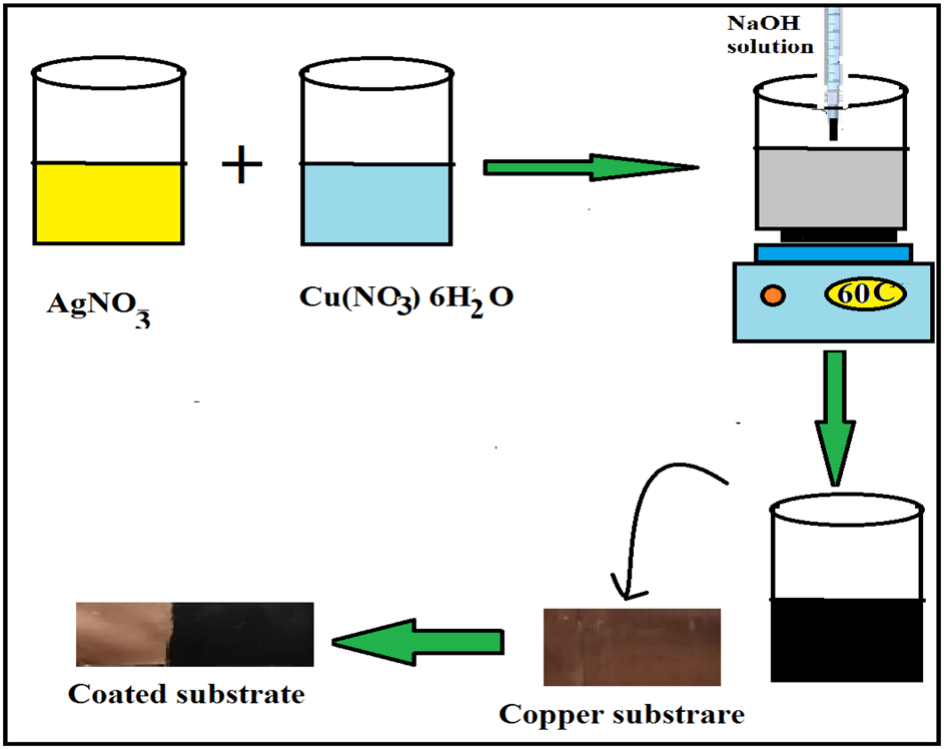
Schematic illustration of perpetration process of Ag-CuO/epoxy coating.

Ag-CuO NPs were synthesized through the cost-effective co-precipitation method. In this process, equimolar quantities (1:1) M of Cu (NO_3_)_2_0.6H_2_O, and AgNO_3,_ were individually dissolved in 10 ml of deionized water and thoroughly mixed for 20 min using a magnetic stirrer. Subsequently, a 2 M solution of NaOH was introduced into the mixture. The resulting blend was stirred for additional hour at 60 °C, leading to the formation of black colored Ag-CuO NPs as illustrated in Fig. 1. The precipitated powder was subjected to multiple washes with ethanol and deionized water, followed by drying in an oven 80 °C for 1 h. Subsequently, it was annealed for 4 h in an electric furnace furnce at 600 °C with a heating rate of 4 °C/min. To ensure the purity of obtained powder, X-ray diffraction (XRD) analysis was performed.

### Coating samples

Various percentages of Ag-CuO powder (0, 0.5, 1.0, 2.0, and 5.0 wt%) were dispersed into the resin under vigorous stirring, ensuring a uniform distribution of the NPs within the epoxy coating to prevent agglomeration, which can reduce the barrier effect^32^. This process is illustrated in Fig. 1. Subsequently, the hardener of (1/3) of the resin volume was added to the nanoparticles-resin mixture and mechanically stirred. Copper sheets were coated by a layer of the prepared nanocomposite, consisting of both pure epoxy resin and (Ag-CuO/epoxy) in various ratio. The resulting coatings were applied to copper substrate using the solution casting technique.

**Figure 2:**
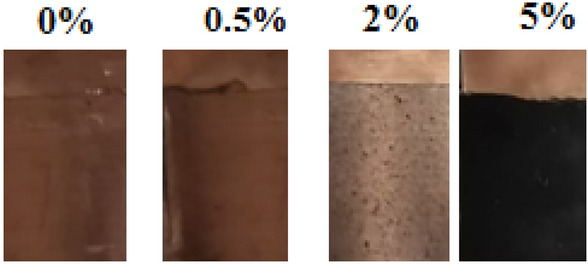
Photographic image of copper coated by 0, 0.5, 2.0, and 5.0 wt%) Ag-CuO/epoxy hybrid nanocomposite.

The prepared hybrid nanocomposite samples were allowed to dry and harden at room temperature for 72 h. Afterward, They were washed with deionized water to eliminate any contaminants and ensure throughout solidification. In Fig. 2, a photographic image display copper substrate coated with varying concentration (0, 0.5, 2.0, and 5.0 wt%) Ag-CuO/epoxy hybrid nanocomposites.

### Characterization

The composition and the crystallinity of the prepared NPs and the synthesized nanocomposites were investigated using a Bruker, D8 Advance, X-ray diffractometer **(**XRD) with Cu-Ka radiation at 40 kV. The analysis was conducted in the 2θ range of 10–80° with a scanning rate of 0.02^0^/min step.

FT-IR instrument (Perkin Elmer) operating in the range of 4000–400 cm^−1^ was utilized to identify the chemical composition and functional groups of the investigated Ag-CuO/Epoxy of the investigated samples.

High-resolution transmission electron microscopy (HRTEM) was performed using a JEM-ARM300F instrument operating at 200 kV to investigate the crystallographic structure of Ag-CuO NPs.

Field Emission Scanning Electron Microscopy (FESEM) was conducted using a JEM-ARM300F high resolution scanning electron microscope operated at 200 kV was used to study the topographical structure of the prepared Ag-CuO/Epoxy hybrid nanocomposite film coated on the copper surface.

Additionally, the hardness of the Ag-CuO/Epoxy hybrid nanocomposite was determined using Shimadzu, Type M hardness testing machine from Japan.

The evaluation of surface wettability on copper substrate, including with 0%, and 5% hybrid nanocomposite, was conducted by measuring the Contact angles (θ). This measurement was performed using the (OCA 15EC) instrument, 70794 Filderstadt, Germany.

### Electrochemical measurements

Electrochemical measurements were conducted using electrochemical Auto lab Potentiostat/Galvanostat 'PGSTAT 302N'. A three-electrode cell setup was utilized for these measurements. The working electrode, which had an exposed surface area of 1 cm^2^, consisted of both uncoated and coated copper substrates with active material. The reference electrode used was Ag/AgCl, while platinum served as the counter electrode. The assessment of corrosion protection performance was carried out in an aqueous solution containing 3.5 wt% NaCl solutions, simulating a marine environment, this assessment included measurements of OCP, PDP, and EIS measurements.

#### Open circuit potential (OCP)

The uncoated and coated copper substrates were immersed in 3.5 wt% NaCl solutions for duration of 1 h. During this immersion, alterations in the OCP of the copper substrate including those coated with various concentrations (0, 0.5, 1.0, 2.0, and 5.0 wt%) of Ag-CuO/epoxy hybrid nanocomposites, were monitored. This allowed for an assessment of the impact of the coating on copper corrosion.

#### Potentiodynamic polarization (PDP)

Both uncoated and coated substrates were immersed in 3.5 wt% NaCl solutions until constant potential values were achieved (OCP). The Potentiodynamic polarizations were conducted by varying the potential ± 250 mV from OCP with a scan rate of 1 mVs^−1^. From the resulting Tafel curves, corrosion parameters were derived including corrosion current density “I_corr_”, corrosion potential “E_corr_”, and polarization resistance “R_pol_”. To assess the protective effectiveness of the coating, the following formula was used for calculation:

1 $$ PE\% = \frac{{I_{o} - I_{c} }}{{I_{o} }} \times 100 $$

where, I_o_ and I_c_ are the corrosion current densities without and with coating, respectively.

The coating porosity was calculated using the polarization resistance (R_pol_) with the following equation:

2 $$ P = \frac{{R_{puc} }}{{R_{pc} }} \times 10^{{ - \frac{\Delta E}{{\beta \alpha }}}} $$

where, R_puc_ and R_pc_ denote the polarization resistance of the uncoated and coated substrates, respectively. ∆E_corr_ represents the difference between the corrosion potentials of uncoated and coated substrates. β_a_ is the anodic Tafel slope for the uncoated substrates.

#### Electrochemical impedance spectroscopy (EIS)

EIS measurements were conducted on both uncoated and coated copper surface by applying a 10 mV amplitude AC signal in the frequencies range 0.01 Hz–50 M Hz at OCP. The collected data were analyzed using Nova1.11 software.

## Results and discussion

### Characterization

#### X-ray diffraction (XRD)

**Figure 3:**
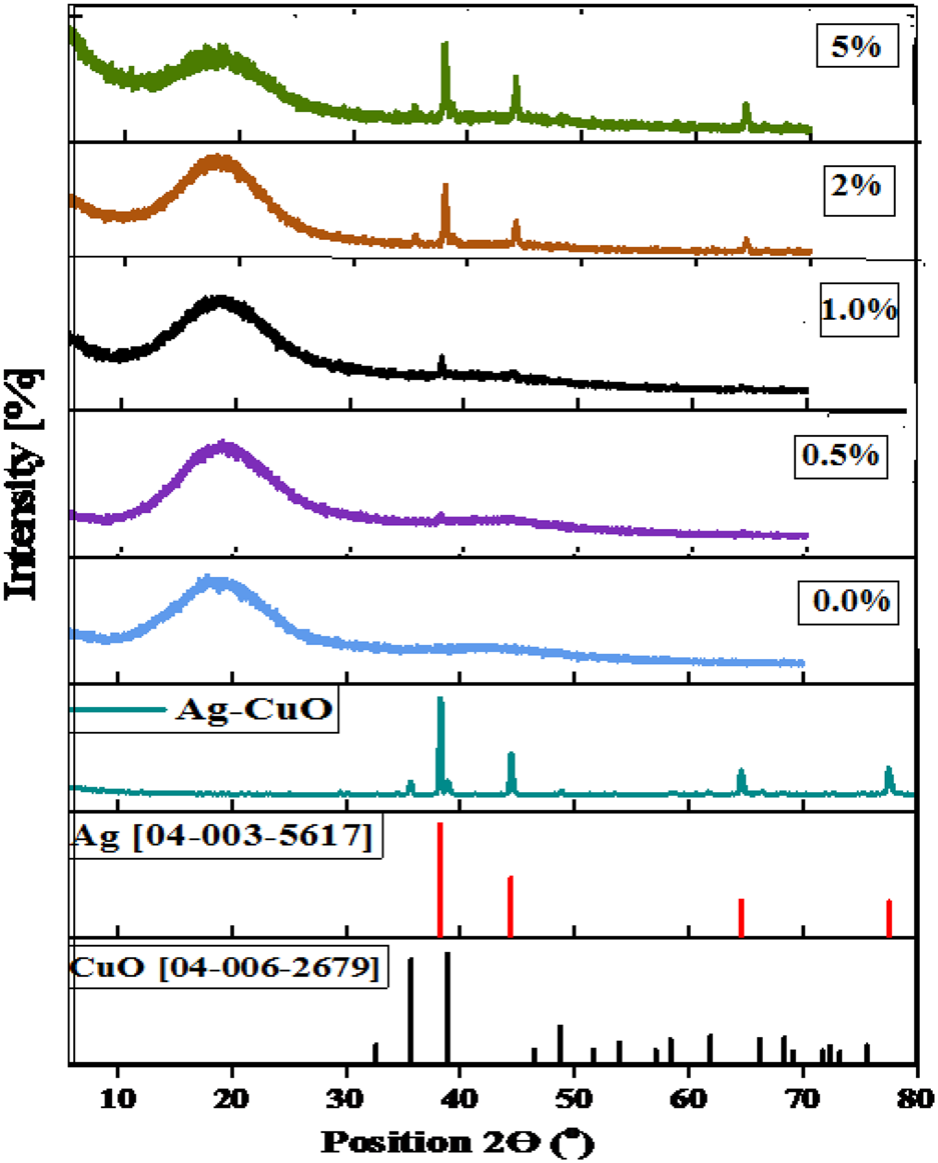
XRD spectra of Ag-CuO NPs, pure epoxy (0%), and (0.5, 1, 2 and 5 wt%) Ag-CuO/epoxy hybrid nanocomposites.

Figure 3 illustrates the XRD pattern for Ag-CuO NPs and the prepared Ag-CuO/epoxy composite with different Ag-CuO wt%. The XRD patterns for all the examined samples are compared with the corresponding ICDD cards. From the figure, it is evident that the investigated NPs closely match the ICDD card [04-006-2679] for the CuO monoclinic phase and [04-003-5617] for the Ag cubic phase. The diffraction peaks indicate the formation of a single phase. Peaks at 2θ = 38.5°, 44.4°, 65° and 77.4°; are ascribed to (111), (200), (220), and (311) planes of silver with face-centered-cubic phase. Furthermore, peaks at 2θ = 35.5°, 38.9° and 48.8° are related to the (002), (200), and (−202) planes of the CuO monoclinic phase^33^. The average crystal size of Ag-CuO, calculated using the Scherrer equation, is approximately 46 nm was calculated using the Scherrer equation^34^

3 $$ D = \frac{0.9\lambda }{{\beta \cos \theta }} $$

where λ = 0.154 nm, β is the full width at half maximum and θ is Bragg’s angle.

As shown in the figure, a broad peak at 2θ = 18.8° corresponds to amorphous epoxy resin. This peak becomes weaker as the concentration of the Ag-CuO NPs increases due to the intercalation of Ag-CuO NPs within the epoxy resin matrix. It is noticeable that the crystallization becomes increasingly evident in the investigated samples as the increasing the concentration of Ag-CuO NPs increases, indicating the homogenous dispersion of NPs within the epoxy matrix.

#### High-resolution transmittance electron microscope (HRTEM)

**Figure 4:**
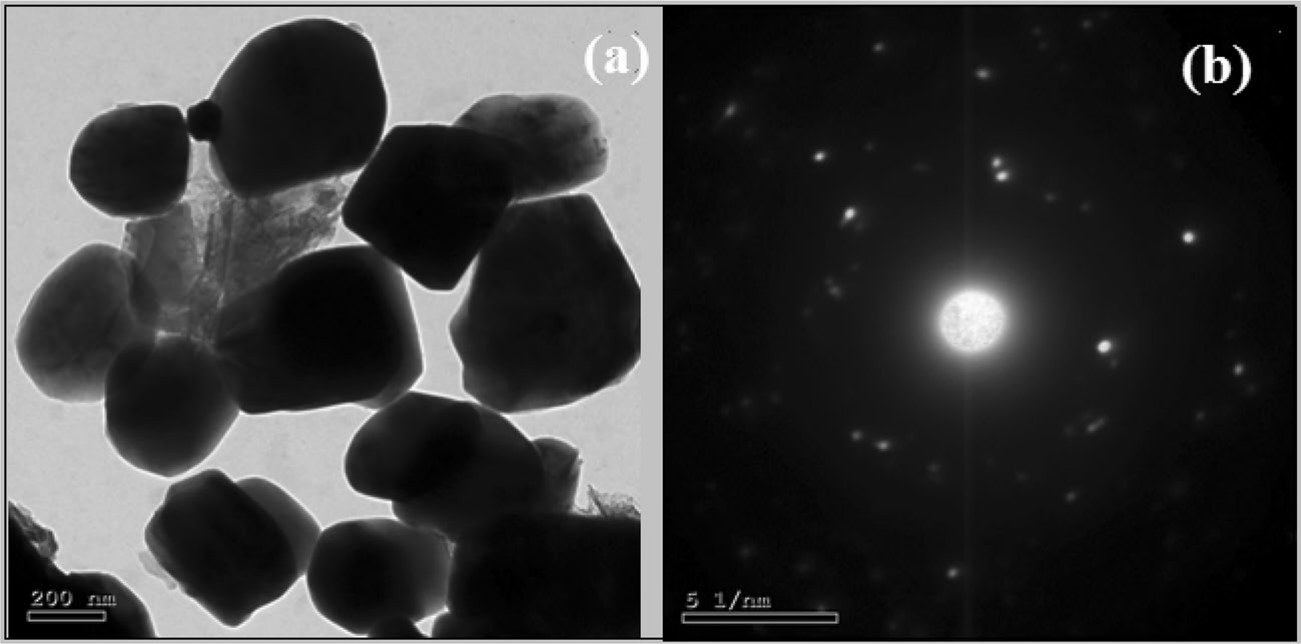
(**a**) HRTEM, (**b**) diffraction fringes of Ag-CuO NPs.

Figure 4a shows the HRTEM of Ag-CuO NPs, for a few agglomerating NPs in the nanoscale. Figure 4b shows uniform diffraction fringes, indicating the crystalline properties of investigated NPs.

#### Fourier-transform infrared spectroscopy (FT-IR)

**Figure 5:**
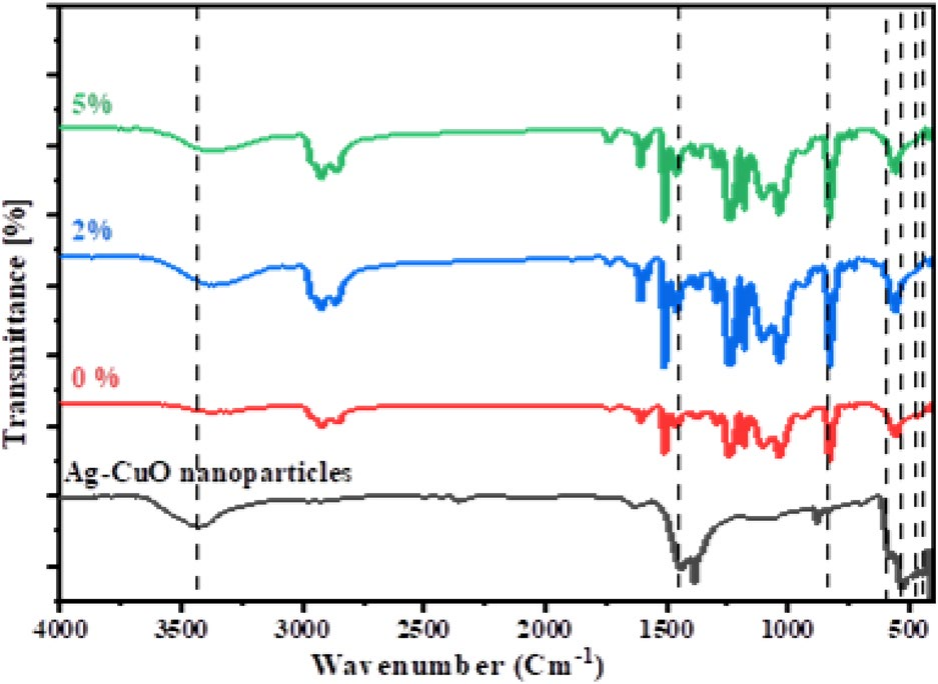
FTIR spectra of Ag-CuO NPs, pure epoxy (0%) and 2.0, 5.0 wt% Ag-CuO/epoxy hybrid nanocomposite.

Figure 5 displays the FTIR spectra of the synthesized and epoxy samples filled with different ratios of Ag-CuO NPs. In these spectra the peak are around 3424 cm^−1^ observed in both Ag-CuO NPs, and epoxy samples filled with the NPs signify the –OH vibration mode of water, which could be adsorbed on the surface of the NPs.

The peaks at 1398 cm^−1^ and 878 cm^−1^ are attributed to the Ag–O stretching vibration mode^35^. Additionally, The vibrations detected at 443 cm^−1^ and 535 cm^−1^ represent Cu–O stretching and vibration modes specific to the monoclinic phase of CuO^36^. The peaks in the FT-IR spectrum correspond to Ag-CuO NPs and the Ag-CuO/epoxy composite.

FT-IR spectra of hybrid nanocomposite containing (0, 2, and 5%) Ag-CuO NPs reveal various peaks corresponding to functional epoxy groups. Vibrations at 2850 cm^−1^ and 2919 cm^−1^ are attributed to C–H stretching bond, while the band at 1425 is related to the C–H bending mode. Additionally, absorption bands in the 1100–1000 cm^−1^ indicate C–H deformation. The epoxy samples under study exhibit a strong band at 1035 cm^−1^ which corresponds to the C–O–C groups. Bands at 553–633 cm^−1^ correspond to the C–O–O function group, while bands at 1442 cm^−1^ are associated with the C–C stretch mode.

#### Contact angle (CA)

The contact angle (CA) value is determined by measuring the tangent of the water droplet at the contact point of the three-phase boundaries (solid, liquid, and gas). The average contact angle values were obtained from measurements taken at various locations with three measurements conducted independently.

**Figure 6:**
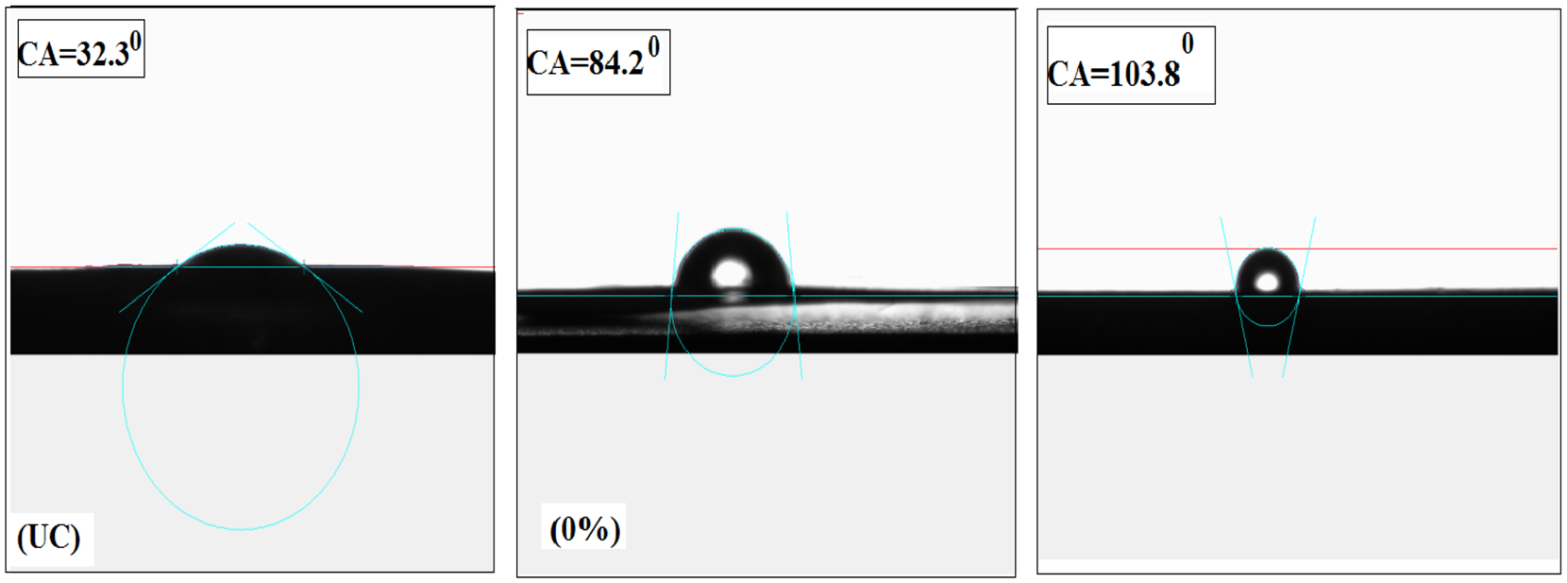
The contact angle (CA) of uncoated surface (UC), neat epoxy coated (0%), and 5% Ag-CuO/epoxy on copper substrate.

Figure 6 displays the CA measurements; it is evident that the uncoated surface (UC) exhibits hydrophilic with CA of approximately 32.3 ± 2°. In contrast, epoxy without NPs demonstrates a less hydrophilic nature; with a CA of approximately 84.2 ± 2°. However, the 5% Ag-CuO/epoxy nanocomposite exhibits a significantly higher CA, measuring around 103.8 ± 1° when compared to the 0% sample. This increase in CA suggests reduced wettability and enhanced hydrophobic characteristics preventing water accumulation and improving self-cleaning properties. The increase in the hydrophobicity for hybrid coatings can be attributed to the effective dispersion and homogeneity of Ag-CuO NPs in the epoxy matrix^29^.

#### Mechanical properties (Hardness)

**Figure 7:**
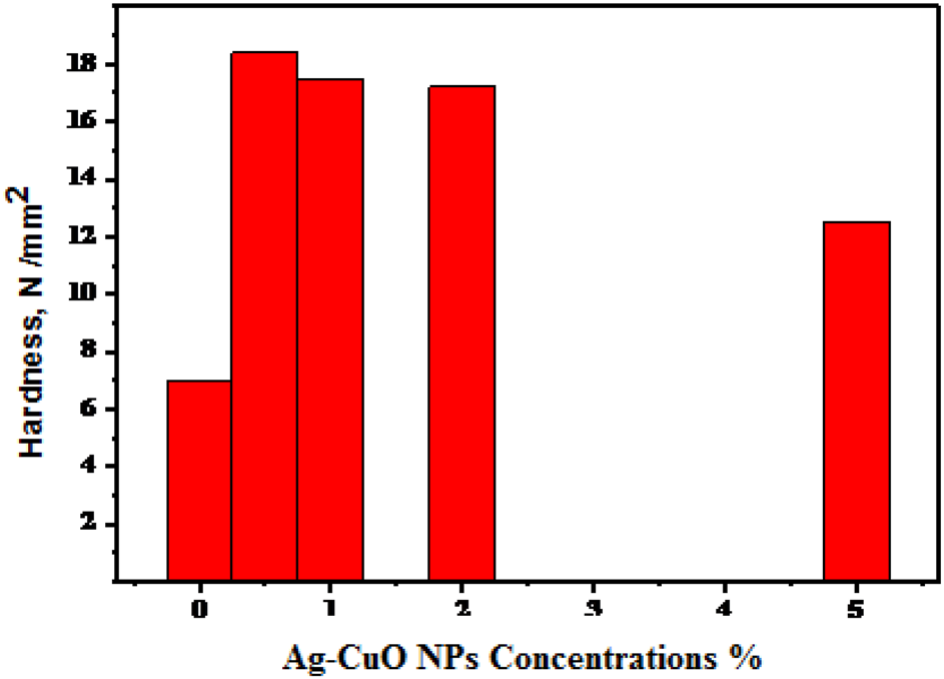
The variation of hardness with different concentrations of Ag-CuO/epoxy coating on copper substrate.

The use of NPs in epoxy composites has been shown to improve the mechanical properties of the material. It has been demonstrated that low concentrations of Ag-CuO NPs increase the epoxy composite's hardness by 2.5 times that of neat epoxy. The change in the hardness of the epoxy composite with the variation of Ag-CuO NPs content is shown in Fig. 7.

Due to proper dispersion of Ag-CuO NPs, the hardness factor is observed to be increased in the presence of NPs. A low concentration of 0.5% shows a larger hardness compared to 5% NPs. The hardness is shown to rise for the 0.5% filler, while it is seen to decrease with greater NP s filler content. It is found that with the increase in filler content, the cluster of Ag-CuO NPs increases and a decrease in hardness is observed and enhanced ductility^37^.

### Electrochemical measurements

Electrochemical measurements were conducted on both uncoated and copper substrate coated with various concentrations of Ag-CuO/epoxy hybrid nanocomposite. These measurments utilized different techniques, including OCP, PDP, and EIS to assess the protective efficiency of the coating.

#### Open circuit potential (OCP)

**Figure 8:**
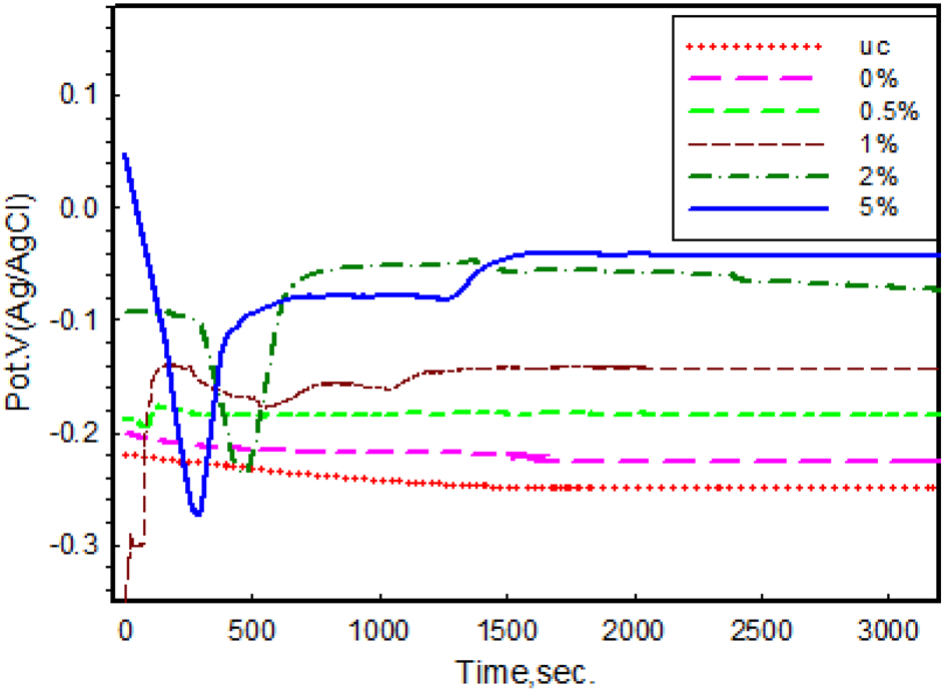
OCP of uncoated copper (UC) and coated with different concentrations (0, 0.5, 1.0, 2.0, and 5.0 wt%) Ag-CuO/epoxy nanocomposite.

Figure 8 illustrates the OCP of uncoated (UC) and the coated copper in NaCl solution for 1 h. A significant change was observed in the behavior of the copper substrate before and after coating. The bare copper exhibits a gradual shift in potential toward the cathodic direction^38^. The curves indicate that the 15 min were sufficient to reach a steady-state potential.

The OCP of coated copper shifted towards positive values compared to the uncoated surface. The steady OCP values for the uncoated copper substrate, pure epoxy (0%), and 5.0 wt% concentrations were 0.248, − 0.225, and − 0.042 V, respectively. From a thermodynamic perspective, the less negative E_corr_ value leads to a lower tendency for corrosion^39^. The 5.0 wt% coat is the most efficient concentration for protection due to the highest positive potential shift. The epoxy coating isolates the copper metal by reducing the permeability of the electrolyte from the surrounding environment^40,41^.

#### Potentiodynamic polarization studies (PDP)

**Figure 9:**
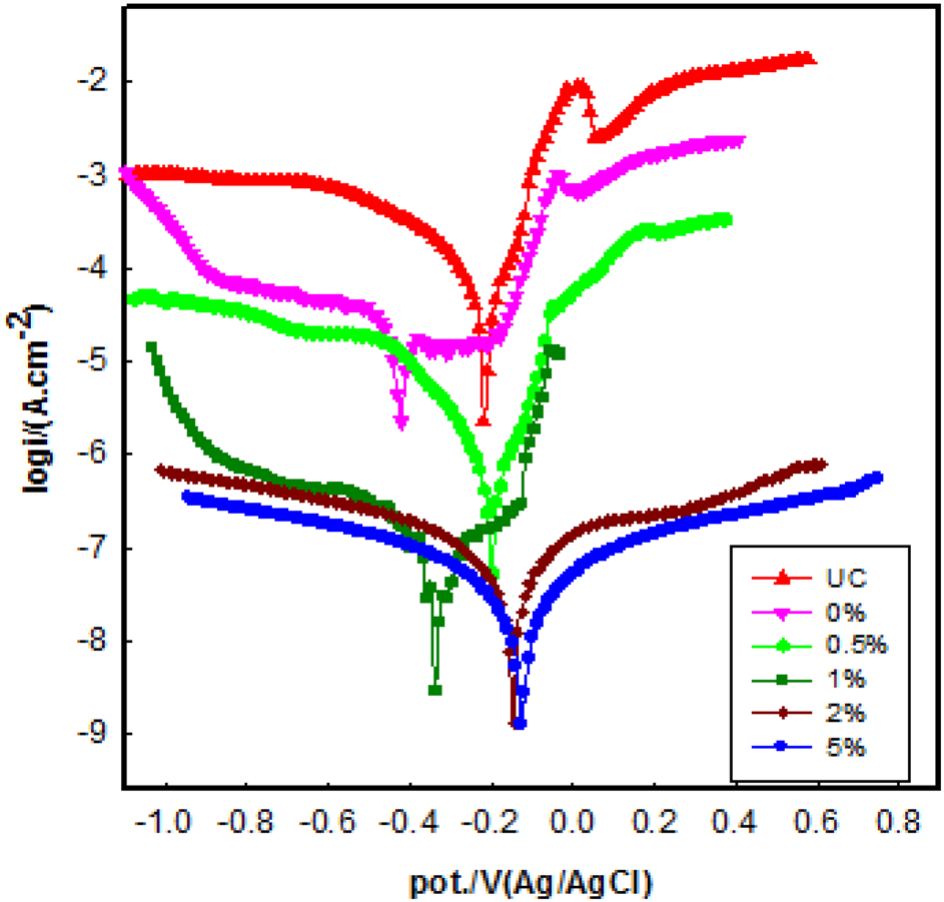
The polarization curves of uncoated (UC) and coated copper with different concentrations in 3.5 wt% NaCl solutions.

Figure 9 displays the PDP curves of uncoated (UC) and coated copper with different concentrations (0, 0.5, 1.0, 2.0, and 5.0 wt%) of Ag-CuO/epoxy hybrid nanocomposite after 1 h of immersion in 3.5 wt% NaCl solution. These data indicate that concentrations 2.0 and 5.0 wt% shift the corrosion potential toward a noble direction. Similar to the OCP, a higher corrosion potential indicates a lower chance of corrosion. The shift in corrosion potential (E_corr_ vs. Ag/AgCl), from − 0.211 V for UC to − 0.120 V for 5.0 wt% coating decreases the dissolution of the copper substrate, revealing the improved corrosion resistance of the coated substrate. At all coating concentrations, both anodic and cathodic current densities shift toward lower values compared to uncoated copper.

The Tafel extrapolation method was used to estimate the corrosion parameters, as listed in Table 1. From the table, the highest current density was 1.02 × 10^−5^ A cm^−2^ for UC and the lowest was 3.29 × 10^−8^ A cm^−2^ for the 5.0 wt% coated copper substrate. As a result of this remarkable reduction in corrosion current, the corrosion protection was enhanced by decreasing the corrosion rate (CR). The corrosion rate of the 5.0 wt% Ag-CuO/epoxy hybrid nanocomposite is 3.81 × 10^–4^ mmy^−1^ which represents more than a 99% reduction in CR compared to UC and the pure epoxy coating. Another important parameter is the polarization resistance (R_Pol_) which identifies the coating's ability to prevent electron exchange in a corrosive environment.

**Table 1:** The polarization parameter values for uncoated and different concentrations of epoxy coated copper in NaCl solution from PDP at 25 ± 0.2 °C.

Conc., wt%	E_corr._, V(Ag/AgCl)	β_a_, Vdec^1^	β_c_, Vdec^1^	I_corr_, A cm^−2^	R_P_, K Ohm m^2^	C.R, mmy^−1^	SD	PE, %	P, %
UC	− 0.211	0.301	0.095	7.73 × 10^–5^	0.407	0.895	1.5 × 10^–4^	–	–
0	− 0.416	0.144	0.193	1.02 × 10^–5^	3.51	0.118	1 × 10^–4^	86.76	1.10
0.5	− 0.209	0.261	0.921	8.46 × 10^–6^	10.43	0.098	6 × 10^–4^	89.60	1.70
1.0	− 0.333	0.410	0.258	8.36 × 10^–8^	822.6	9.67 × 10^–4^	2 × 10^–5^	99.75	0.08
2.0	− 0.159	0.609	0.626	7.87 × 10^–8^	1704.0	9.10 × 10^–4^	1 × 10^–5^	99.80	0.02
5.0	− 0.120	0.485	0.553	3.29 × 10^–8^	3404.6	3.82 × 10^–4^	1 × 10^–5^	99.90	0.01

As seen in Table 1, R_pol_ increased from 0.406 kΩ cm^2^ for uncoated copper to 3404 kΩ cm^2^ for a 5.0 wt% coated copper substrate. The highest value indicates its superior resistance to diffusive aggressive ions such as chloride. Finally the protection efficiency, calculated using the corrosion current density with Eq. (1), reached a high value of 99.8% and 99.9% for 2.0, 5.0 wt% Ag-CuO/epoxy hybrid nanocomposite coatings.

**Figure 10:**
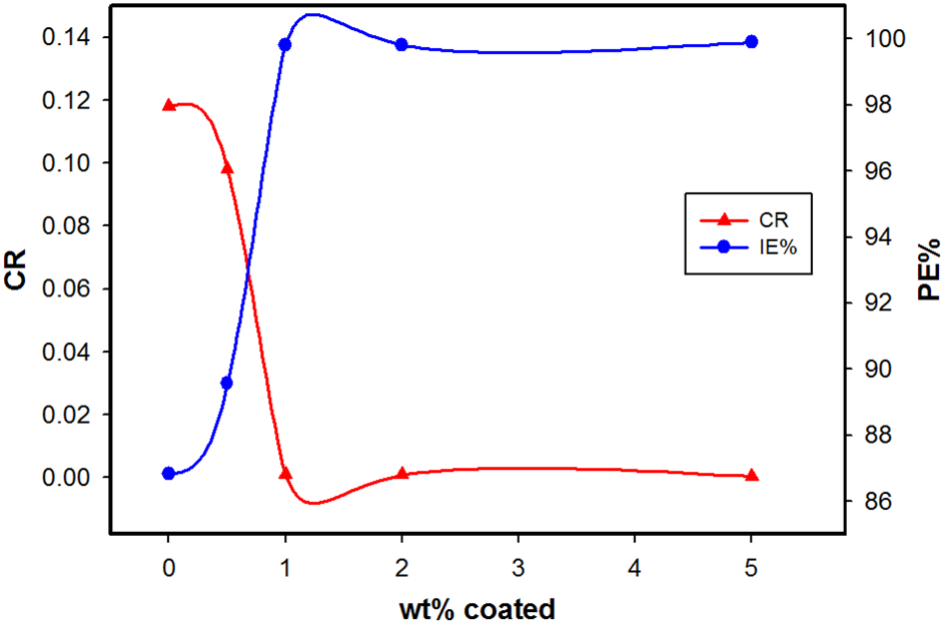
The variation of corrosion rate (CR) and protection efficiency (PE %) with wt% epoxy hybrid nanocomposite coating.

The variation of corrosion rate (CR) and the protection efficiency (PE) with wt% of Ag-CuO NPs in the epoxy coating is shown in Fig. 10. It is evident that the addition of Ag-CuO NPs in the epoxy coatings decreased the corrosion rate and increased the anticorrosive efficiency of the coating on the copper surface.

On the other hand, the improved corrosion protection provided by the Ag-CuO/epoxy hybrid nanocomposite coating over the protection offered by epoxy alone may attributed to the dispersion of Ag-CuO NPs in the epoxy matrix, which increases the tortuosity of the pathways of oxygen and chloride corrosive agents^5,42,43^. These NPs create conductive pathways that prevent the localization of electrons, reducing the rate of oxygen reduction at the coating/metal interface and, consequently, reducing the likelihood of coating delaminating^27^.

The coating porosity is another vital parameter that affects the anticorrosive behavior of the epoxy coating. Porosity was calculated from potentiodynamic polarization resistance (R_pol_) using Eq. (2) with the anodic Tafel slope of β_a_ set at 0.301 Vdec^1^ for the UC copper substrate^44^. The calculated porosity values are listed in Table 1.

As seen, P% decreased from 1.10 to 0.01 for 0% and 5.0 wt% hybrid nanocomposite coatings, respectively. Generally, the pure epoxy (0%) has many pores, which provide an easy pathway for corrosive ions to penetrate. but the presence of Ag-CuO NPs of various wt% may block these pores, resulting in enhancement of coating homogeneity and, hence, improved corrosion protection.

The lower porosity values improve resistance by reducing the penetration of the aggressive ions to reach the copper surface^45^. Ag-CuO NPs create a uniform structure on the epoxy coating providing longer pathways for aggressive ions. After the water and electrolyte penetrate the coating, the anticorrosive NPs begin to passivate the surface^46^.

**Figure 11:**
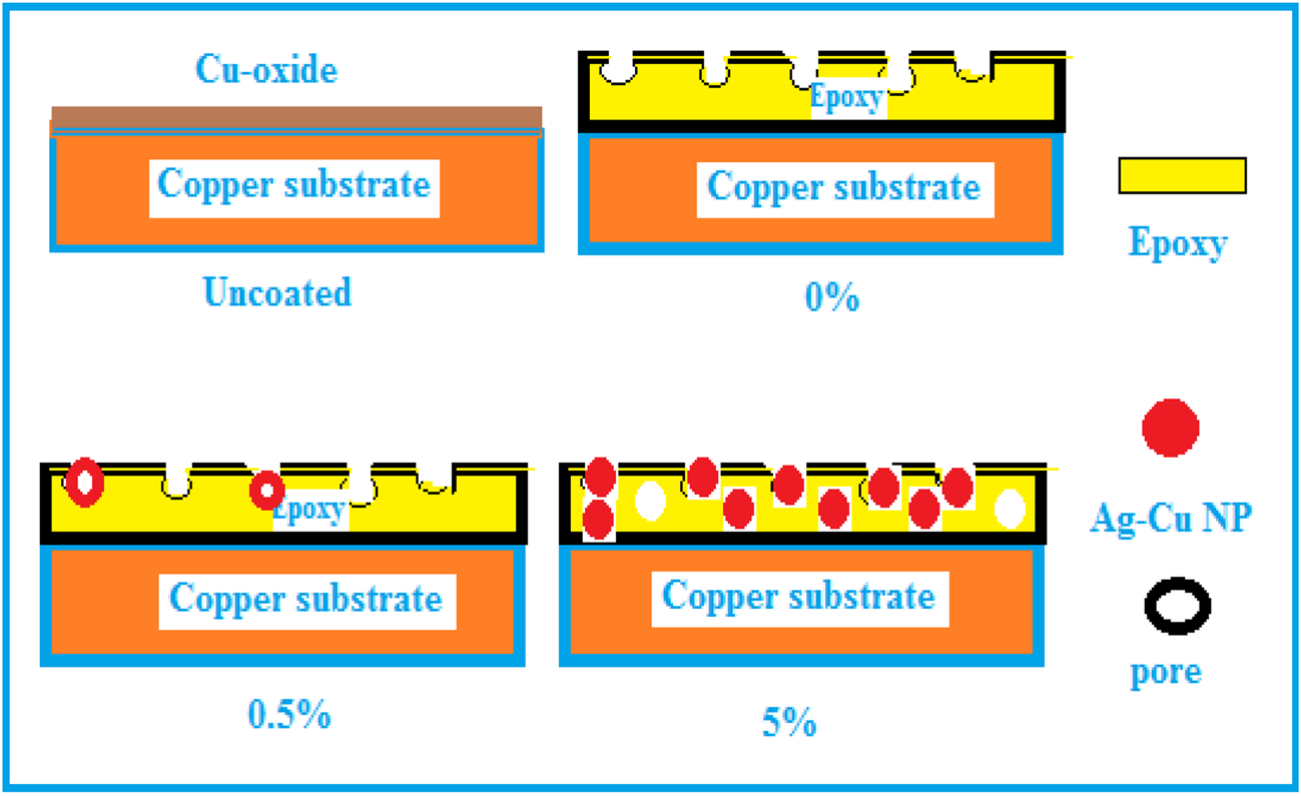
Schematic illustration of the filling of Ag-CuO NPs on the epoxy pores**.**

The presence of NPs in epoxy matrix induces a healing effect by filling holes and voids, creating a strong barrier and high protection efficiency^47^. According to the results, 5.0 wt% Ag-CuO NPs have uniformly dispersed into the epoxy coating, filling the pores, and acting as a physical barrier against corrosive agents as seen in Fig. 11. Therefore, NPs restrict the penetration of corrosive species in the coating/metal interface. Hence 5.0 wt% is considered the optimum coating concentration, as evidenced by a lower porosity, lower current density, and enhanced corrosion resistance and protection efficiency.

The obstructive impact of NPs within epoxy coatings lies in their ability to impede the advancement of corrosion. They achieve this by acting as a barrier, obstructing the ingress of corrosive substances like chloride ions and other oxidizing agents through the interface between the coating and the copper substrate. This phenomenon is primarily attributed to the heightened hydrophobic characteristics of the coating surface^30^.

#### Electrochemical impedance spectroscopy (EIS)

**Figure 12:**
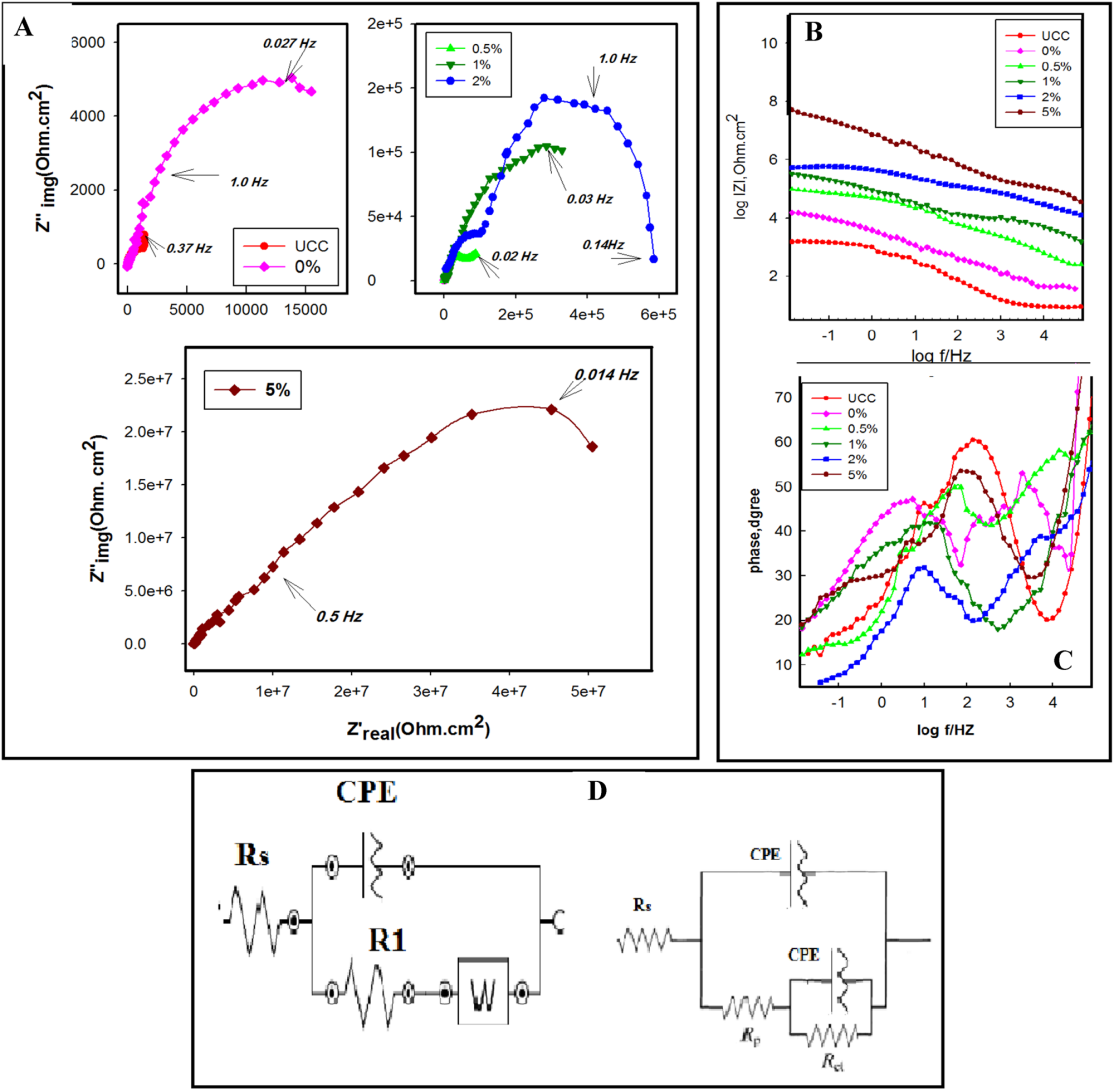
Nyquist plots (**A**), Bode plots (**B**), Phase plots (**C**), and (**D**) equivalent electrical circuit fitting the EIS data.

The degree of corrosion resistance was assessed using non-destructive EIS at the open circuit potential. The Nyquist plots of uncoated and coated copper with different concentrations of Ag-CuO/epoxy hybrid nanocomposite (0, 0.5, 1.0, 2.0, and 5.0 wt%) in 3.5 wt% NaCl solution are depicted in Fig. 12A. From the figure, it can be observed that the diameter of the UC is the smallest, and the diameter increases with increasing wt% Ag-CuO/epoxy hybrid nanocomposite coating. The largest diameter attributed to the 5.0 wt% Ag-CuO/epoxy hybrid nanocomposites coating, indicating that it effectively prevents the penetration of corrosive species, leading to higher resistance.

Impedance modulus (log |Z|), and phase angle (θ) are plotted against log frequency representing Bode and Phase plots as shown in Fig. 12B,C, respectively. At high frequencies the solution resistance is dominant, while at low frequency, the coating resistance is dominant. The middle frequency range represents the capacitive region. The Bode plot of 5.0 wt% shows a high |Z| at the high frequency region indicating the excellent barrier^48^.

These anticorrosion properties can be credited to the dispersion of the prepared Ag-CuO NPs in epoxy which reduces the rate of water and oxygen penetration.

The phase plot shown in Fig. 12C demonstrates a shift in phase angle to low frequency for pure epoxy and subsequent shift to high frequency with higher wt% indicating high corrosion protection. EIS plots were modeled by the best fit equivalent circuit as shown in Fig. 12D. The fitting quality of the suggested circuit has been verified by the chi-square (*χ*^2^) values listed in Table 2. A smaller χ^2^ value indicates a better fit of the recommended equivalent circuit, and fit results match well with the experimental data.

**Table 2:** The EIS parameters values for uncoated and different concentrations of epoxy coated copper in 3.5% NaCl solution.

Samples	wt%	Rs (Ω)	R_c_ (K Ω cm^2^)	CPE_c_ Yo (Ω^−1^ cm^−2^S^n^)	n	*R*_*ct*_ (KΩcm^2^)	*CPE*_*dl*_ Yo (Ω^−1^ cm^2^S^n^)	n	w	χ^2^
UCS	–	6.2	1420	1.76 × 10^–4^	0.586	–	–		5.6 × 10^–3^	0.09
Blank	0	8.9	0.350	5.77 × 10^–4^	0.610	10	2.95 × 10^–4^	0.592	–	0.16
Ag-CuO	0.5	11.8	0.340	1.32 × 10^–6^	0.800	89	3.94 × 10^–6^	0.561	–	0.19
	1.0	12.6	416	3.85 × 10^–6^	0.541	71	1.55 × 10^–8^	0.825	–	0.022
	2.0	10,243	500	3.87 × 10^–7^	0.516	30	8.22 × 10^–10^	0.987	–	0.017
	5.0	21,871	64,000	5.27 × 10^–8^	0.750	6900	1.12 × 10^–8^	0.820	–	0.012

The parameters listed in Table 2 include the electrolyte resistance (R_s_) and the charge transfer resistance (R_ct_) at the copper coating interface^49^. Warburg impedance (Z_w_) accounts for the diffusion of Cu^2+^ from the surface to the bulk solution and the diffusion of dissolved oxygen from the bulk solution to the copper surface^50^, R_c_ is the coat resistance due to the ion conducting paths across the coating. The constant phase element (CPE) was used instead of the ideal double layer capacitance.

4 $$ Z_{CPE} \left( \omega \right) = \left[ {Q\left( {j\omega } \right)^{n} } \right]^{ - 1} $$

Where, Q is the constant phase element, ω is the angular frequency in radians, j is the imaginary number, and n is the exponent.

The measured value of |Z| (Table 2) was found to be a high value 7.7 kΩcm^2^ for the epoxy coating with 5.0 wt% Ag-CuO NPs. The capacitance of the coating is an important parameter for estimating the coating's integrity as it reflects the degree of water and electrolyte penetration. The capacitance was the lowest for 5.0 wt% indicating an undamaged coating. Conversely, the highest capacitance value for epoxy without Ag-CuO NPs suggests significant great electrolyte penetration.

As presented in the table, pure epoxy (0% NPs) exhibits poor barrier properties; the electrolyte can penetrate the coating film, resulting in low resistance equal to 350 Ohm. The R_c_ values obtained from EIS data increased with increasing wt%; R_ct_ values increased from 1.5 × 10^2^ for UC to 6.9 × 10^7^ for the 5.0 wt% coated sample, indicating that the Ag-CuO NPs exhibited better charge transfer resistance than pure epoxy. This improvement was related to the increased barrier properties, which hindered the diffusion of water, oxygen, and chloride through the epoxy matrix^51^.

The lower value of capacitance 1.1 × 10^–8^ for the 5.0 wt% coated provides further support for the protection of copper substrate^52^. The addition of Ag CuO NPs into the epoxy matrix exhibited better charge transfer resistances than pure epoxy, indicating that the nanoparticles improve the barrier properties of epoxy matrix^53^.The coating resistance (R_c_) is an important parameter that reflects the barrier ability of a coated film in the electrolyte solution.

**Figure 13:**
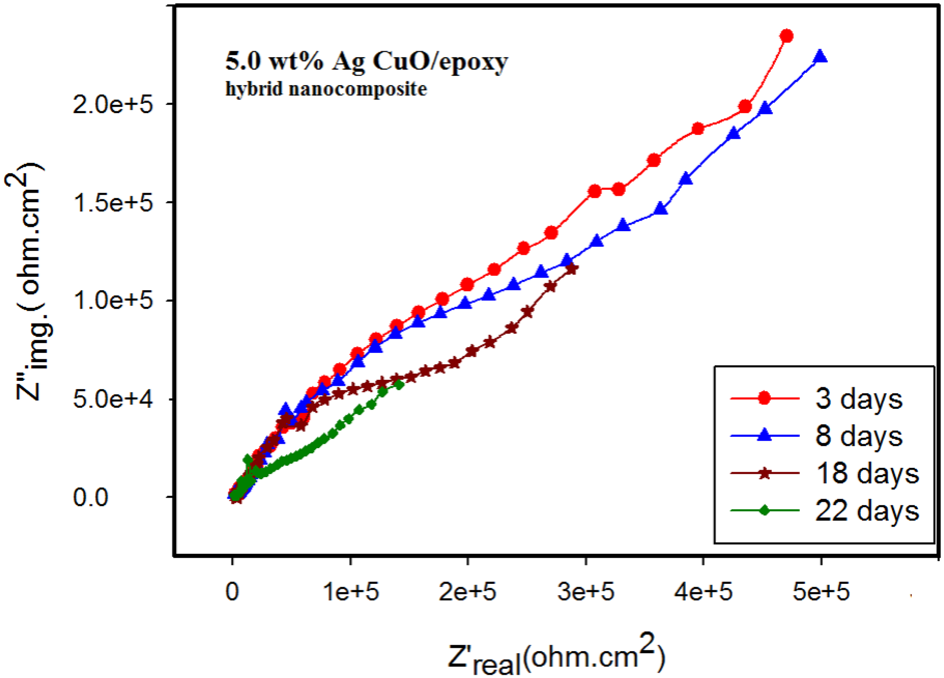
The Nyquist plots at different immersion times for 5.0 wt%.

Hence, the low value of double-layer capacitance and the high value of charge transfer resistance, indicate the excellent corrosion performance of the epoxy hybrid nanocomposite coating. Based on OCP, PDP, and EIS measurements, it was found that the optimum concentration is 5.0 wt% Ag-CuO/epoxy. Therefore, the stability of the coating was studied by recording the EIS with time as shown in Fig. 13.

The Nyquist plot initially displays the highest values, as observed in Fig. 12. As seen in Fig. 13, after 3 days the values slightly decreased but still remained relatively high for up to 18 days. The variation may indicate the gradual penetration of electrolyte through the epoxy coated films, with water and aggressive ions unable to reach the coating/metal interface during this time. However after 22 days, it appears that corrosive ions have reached the coating/metal interface, resulting a decrease in impedance^54,55^. Indeed, the higher impedance values observed in the epoxy hybrid composite coatings after immersion serve as evidence of their performance in protecting copper against corrosion.

### Scanning electron microscope (SEM)

**Figure 14:**
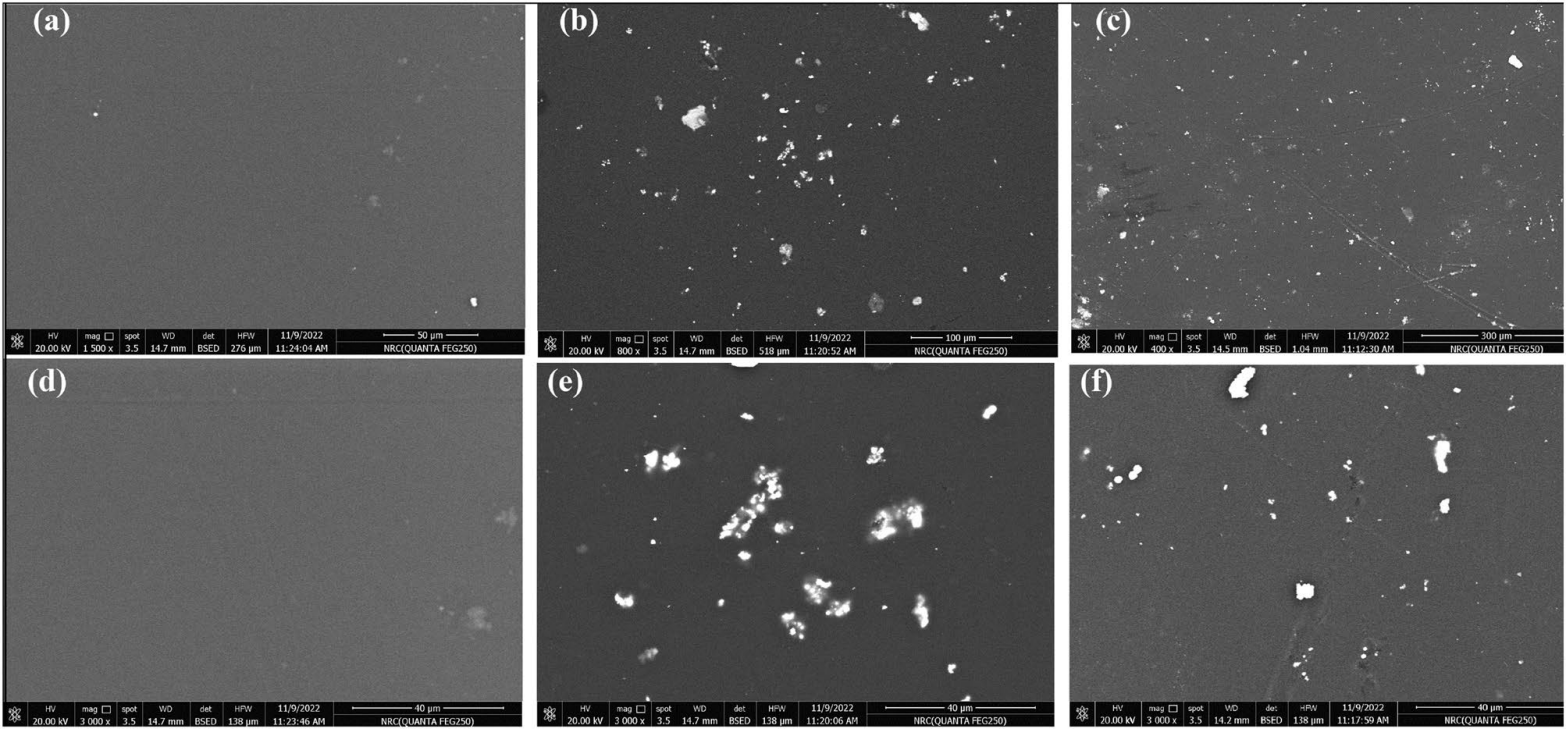
SEM of coated epoxy (**a**) 0%, (**b**) 5% before immersion, (**c**) 5% after immersion at low magnification, and (**d**–**f**) respectively at the high magnification.

SEM analysis of the pure epoxy and AgCuO/epoxy coatings on copper substrates, before and after immersion in 3.5% NaCl solution was conducted to assess their morphological changes. Figure 14a,d displays SEM image of the unexposed epoxy sample (0%) at different magnification, revealing a uniform and smooth coating without any defects. Figure 14b,e change in the morphological structure is observed for 5% Ag CuO/epoxy hybrid nanocomposite. It shows that the NPs are well-dispersed; resulting in a uniform coating without any defects, pores, or cracks in the matrix. Furthermore, there is no evidence of Ag-CuO NPs agglomeration in the epoxy matrix, indicating good dispersion. Figure 14c,f presents SEM image of the 5% Ag-CuO NPs/epoxy coatings after immersion in the aggressive 3.5% NaCl solution, showing no hole or damage, and the coating remains fully intact on the copper substrate.

This phenomenon can be attributed to the high surface area and small size of Ag-CuO NPs (46 nm as indicated by the XRD result). These characteristics reduce the pathways available corrosive electrolyte to penetrate through the coating matrix, thus slowing down the corrosion process enhancing coating resistance^56^.

## Conclusion

This study aimed to develop a novel Ag-CuO/epoxy protective coating on copper substrate. The Ag-CuO NPs were successfully synthesized by a low-cost co-precipitation method. Epoxy was shown as the coating material due to its high safety and excellent adhesion to various substrates. Ag-CuO NPs were dispersed in epoxy at different weight percentages, including 0, 0.5, 1.0, 2.0, and 5.0 wt%. The characterization of the composite coating by FTIR, XRD, SEM, and TEM confirm the uniform distribution and composition of the Ag-CuO NPs within the epoxy matrix.

Regarding the mechanical properties of the coating, the addition of NPs generally enhanced properties such as the hardness. Indeed, the optimal concentration of NPs for improving mechanical properties may differ from that for enhancing anticorrosive properties. The contact angle measurements suggested that the coating containing 5.0 wt% Ag-CuO exhibited a high hydrophobicity of 104°, which prevented the penetration of corrosive ions into the coating.

The corrosion performance of both coated and uncoated copper samples was assessed using various electrochemical techniques, including OCP, PDP, and EIS, in a corrosive environment with a 3.5 wt% NaCl solution. The results from PDP and EIS experiments demonstrated the effectiveness of the Ag-CuO/epoxy hybrid nanocomposite as a protective coating for copper in the presence of NaCl solution.

The study's findings highlighted that the coating with 5.0 wt% Ag-CuO loading demonstrated the greatest highest corrosion resistance compared to the other coatings tested. This was evident from several key observations, including a significant shift in corrosion potential, a reduction in corrosion current density, an impressive coating efficiency of 99.9%, and a remarkably low coating porosity of only 0.01%. Additionally, SEM and EDX analyses of the corroded samples confirmed that the composite coating effectively thwarted the corrosion process by establishing a protective layer on the copper surface. The study also provides valuable insights into how factors like NP loading and hydrophobicity influence the corrosion behavior of composite coatings.

The even distribution of the NPs with the coating and the extension of the pathway for the corrosive ions through the epoxy matrix are the primary factors contributing to the enhanced resistance of the coating. The findings from the electrochemical impedance spectroscopy (EIS) are consistent with the results obtained from Tafel polarization measurements, further supporting the effectiveness of the composite coating in preventing corrosion.

The findings of this study highlight the potential of Ag-CuO/epoxy hybrid nanocomposite coatings as a promising solution for efficiently protecting copper from corrosion in aqueous environments. Coatings offer effective ways to safeguard copper and can also provide self-cleaning properties to its surface.

## Data Availability

All data generated or analyzed during this study are included in this published article.
